# Dissecting the contribution of *Staphylococcus aureus* α-phenol-soluble modulins to biofilm amyloid structure

**DOI:** 10.1038/srep34552

**Published:** 2016-10-06

**Authors:** Patrizia Marinelli, Irantzu Pallares, Susanna Navarro, Salvador Ventura

**Affiliations:** 1 Institut de Biotecnologia i de Biomedicina, Universitat Autònoma de Barcelona, E-08193 Bellaterra, Spain; 2 Departament de Bioquímica i Biologia Molecular, Universitat Autònoma de Barcelona, E-08193 Bellaterra, Spain

## Abstract

The opportunistic pathogen *Staphylococcus aureus* is recognized as one of the most frequent causes of biofilm-associated infections. The recently discovered phenol soluble modulins (PSMs) are small α-helical amphipathic peptides that act as the main molecular effectors of staphylococcal biofilm maturation, promoting the formation of an extracellular fibril structure with amyloid-like properties. Here, we combine computational, biophysical and *in cell* analysis to address the specific contribution of individual PSMs to biofilm structure. We demonstrate that despite their highly similar sequence and structure, contrary to what it was previously thought, not all PSMs participate in amyloid fibril formation. A balance of hydrophobic/hydrophilic forces and helical propensity seems to define the aggregation propensity of PSMs and control their assembly and function. This knowledge would allow to target specifically the amyloid properties of these peptides. In this way, we show that Epigallocatechin-3-gallate (EGCG), the principal polyphenol in green tea, prevents the assembly of amyloidogenic PSMs and disentangles their preformed amyloid fibrils.

Methicillin-resistant *Staphylococcus aureus* (MRSA) strains are involved in hospital acquired “nosocomial” infections such as endocarditis, necrotizing pneumonia and septic shock upon clinical infection^1^,^2^,^3^,^4^. In the last few decades, new hospital- and community-associated MRSA strains have emerged, showing both antibiotic resistance and enhanced virulence and fitness^5^,^6^. Remarkably, *S. aureus* infective ability is tightly associated with biofilm-mediated resistance to host immune response, chemotherapies and disinfectants^7^. Indeed, the National Institute of Health in the USA has stated that more than 80% of microbial infections are somehow linked to biofilm formation. The biofilm extracellular matrix comprises chemically different macromolecules, including proteins, teichoic acids, extracellular DNA (eDNA) and polysaccharides^7^,^8^.

Amyloids are formed by the self-assembly of aggregation-prone proteins and peptides into highly ordered fibrillar macromolecular structures^9^. The formation of amyloid fibrils is associated with the onset of human degenerative pathologies like Alzheimer’s or Parkinson’s diseases^10^,^11^. The high thermodynamic stability of amyloid fibrils and their resistance in the presence of proteases and detergents is exploited for functional purposes by a number of organisms. In particular, different bacterial species use these assemblies to form and strengthen their biofilm matrices^12^,^13^,^14^. In *S. aureus,* the biofilm amyloid component is mainly constituted by small amphipathic peptides, known as phenol soluble modulins (PSMs)^15^,^16^. PSMs are involved in a series of biological functions that are critical for staphylococci pathogenesis^17^,^18^,^19^. These toxic activities are usually associated with the presence of soluble peptide species^20^ and it has been suggested that PSMs self-assembly may be an evolved strategy to regulate the biological activity of these peptides depending on the environmental conditions. In addition, it appears that PSMs provide *S. aureus* biofilms with resistance to various dispersion agents such as Dispersin B, DNAse I, Protease K, and to mechanical stress^15^. Because PSMs are assumed to share conformational and physicochemical properties, it is thought that all of them contribute to biofilm structuration and therefore that they are all able to transition from an initial soluble monomeric state into an insoluble fibrillar assembly. However, the sequential and structural features accounting for this biological activity have not yet been analyzed in detail and, therefore, there is no direct support for this assumption. In this study, we focused on the α-PSM family and dissected the individual ability of their members to form an amyloid scaffold. We show that, despite their sequential and structural similitude, only certain members of the family are able to form ordered amyloid structures and that this property is anti-correlated with their cytotoxicity. The balance between self-assembly and toxic properties is controlled by the specific physicochemical features encoded in the peptide sequences and by their conformation. Moreover, we demonstrate that the main constituent of green tea, Epigallocatechin-3-gallate (EGCG), for which a potent antimicrobial activity has been reported^21^, is indeed able to prevent the assembly of the amyloidogenic α-PSMs and to disentangle their preformed amyloid fibrils.

## Results

### PSMs sequential aggregation propensity

**Figure 1:**
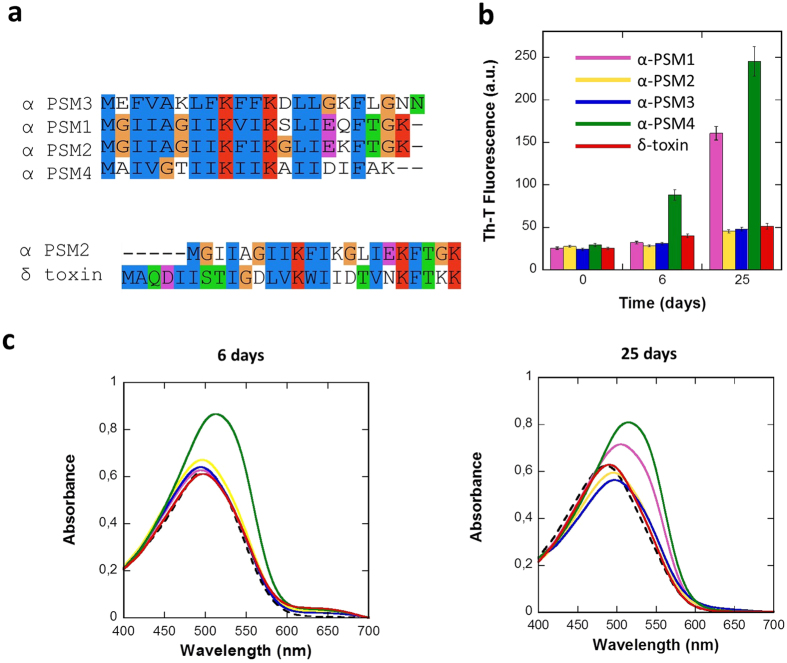
Phenol soluble modulins alignment and *in vitro* aggregation assays. (**a**) Sequences of PSMs are aligned and coloured using ClustalX^23^. (**b**) Aggregation kinetics were monitored by following the change in relative Th-T fluorescence during 25 days. Error bars indicate ± SE (n = 3). (**c**) Congo red (CR) binding was registered at 6 days and 25 days. Free Congo red is represented by a discontinuous black line.

*S. aureus* PSMs are small peptides, which are assumed to adopt amphipathic α-helical structures displaying surfactant-like properties^17^,^18^,^22^. The genes of *S. aureus* PSMs peptides are encoded in its core genome and, accordingly, they are produced by virtually all strains: four are expressed from the alpha operon (α-psm1–4), two are expressed from the beta operon (β-psm1–2), and the delta hemolysin (δ-toxin) is encoded in the regulatory RNA, *RNAIII*^18^. Here we concentrate on the study of α-PSMs and the δ-toxin, because they have been consistently identified as components of the SDS-resistant fraction of *S. aureus* biofilms^15^. α-PSMs consist of 20–22 residues, whereas δ-toxin has 26 residues (Fig. 1a). An alignment of PSMs sequences with ClustalX^23^ (Fig. 1a) shows that they share similar sequential properties, with basic Lys residues conserved at positions 9 and 13 and an hydrophobic Phe conserved at position 18. The hydrophobic character and the amino acid size in the rest of the positions also tend to be conserved. This is not surprising, if we take into account that they are encoded in a single operon, likely arising from gene duplication. In general, hydrophobic aliphatic and aromatic residues dominate α-PSMs composition, accounting for roughly 50% of the sequence. The sequence identity between α-PSMs and δ-toxin ranges 30%, however the positively charged and hydrophobic residues also tend to be conserved (Fig. 1a).

In an attempt to address if, despite their compositional and sequential similitude, PSMs might display different intrinsic aggregation propensities, their sequences were analyzed using the Amylpred2^24^ consensus aggregation prediction algorithm (Table 1). The analysis suggests that the aggregation features of PSMs might differ significantly (Table 1) and, therefore, that these peptides might contribute in a differential manner to *S. aureus* biofilm formation and stability.

### Structural and morphological characterization of PSM aggregation *in vitro*

The ordered β-sheet architecture of amyloid structures can be detected using a range of biochemical and biophysical assays^9^. Because computational predictions suggested that PSMs might differ in their aggregation properties, we decided to characterize experimentally the *in vitro* amyloid-like properties of individual PSMs.

Each PSM peptide was incubated at 200 μM final concentration at room temperature. The aggregation process was monitored during twenty-five days by measuring the binding of the peptides to the amyloid-specific dye Thioflavin-T (Th-T) (Fig. 1b). The fluorescence emission of the Th-T dye is enhanced in the presence of amyloid fibrils^25^,^26^. Only α-PSM1 and α-PSM4 promoted a significant increase in Th-T fluorescence emission at the end of the experiment. The α-PSM4 peptide solution bound Th-T already after 6 days, while α-PSM1 required longer incubation times, indicating a faster aggregation rate for α-PSM4.

In order to confirm the amyloid properties of PSMs peptides we measured their binding to CR. The absorbance of CR increases and the spectrum maximum red shifts in the presence of α-PSM4 after 6 days and in the presence of both α-PSM1 and α-PSM4 after 25 days (Fig. 1d). This spectral change corresponds to that promoted by different amyloid proteins in the aggregated state^27^. No red shift in the CR spectrum was observed in the presence of the rest of PSMs.

**Figure 2:**
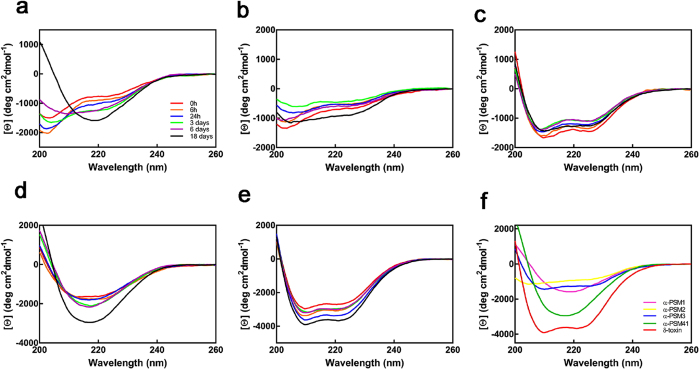
Conformational conversion of PSMs followed by far-UV CD. (**a**–**e**) Evolution of the far-UV CD spectra of PSM peptides during the first 18 days of incubation; (**a**–**e)** panels correspond to α-PSM1, α-PSM2, α-PSM3, α-PSM4 and δ-toxin, respectively. (**f)** Far-UV spectra of PSM peptides after 25 days.

We monitored the conformational conversion of PSMs during the first 6 days of incubation and at the end of the experiment using far-UV circular dichroism (CD) (Fig. 2). Interestingly enough, deconvolution of the CD spectra of freshly dissolved peptides using the CONTIN algorithm^28^ indicated that they display significant differences in secondary structure content at the beginning of the reaction (Table S1). α-PSM3 and δ-toxin exhibited a calculated α-helical content of ≈70%, whereas for α-PSM1 and α-PSM2 it was of ≈30%. The α-PSM4 α-helical content was ≈50%, but the β-sheet component in the spectrum (≈15%) was higher than for the rest of the peptides (≈5%). α-PSM2, α-PSM3 and δ-toxin did not experience any significant conformational change during the first 6 days. In contrast, for α-PSM4 the conversion into a predominant β-sheet structure is already observable after 1 day. However, this peptide solution did not bind Th-T, which suggests that it might contain early oligomeric assemblies. In the case of α-PSM1, a structural conversion into β-sheet containing species is detectable at day 6, despite these conformers did not bind to Th-T yet. After 18 and 25 days, the typical β-sheet signal at ≈218 nm was detected only for α-PSM1 and α-PSM4, in good agreement with Th-T and CR data.

Overall, independently of the used spectral probe, the amyloid-like signature is always higher for α-PSM4 than for α-PSM1 at the end of the reaction and absent for the rest of peptides.

**Figure 3:**
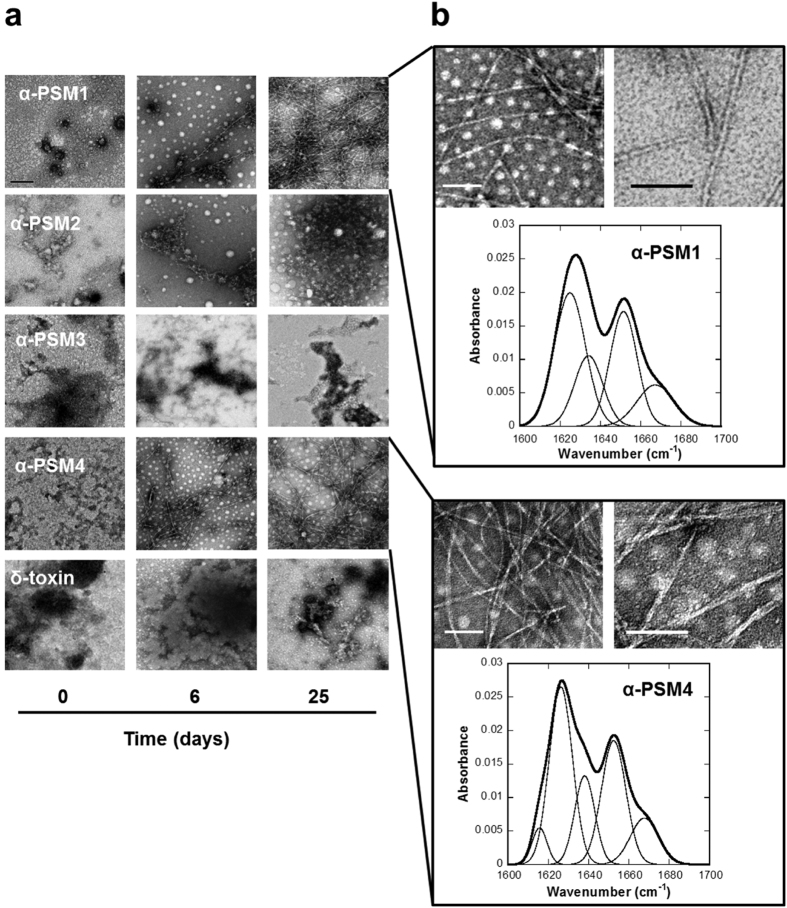
Morphological and structural properties of PSM aggregates. (**a**) TEM micrographs of the aggregates formed by PSMs at different time points. The scale bar represents 0.5 μm. (**b**) α-PSM1 and α-PSM4 fibrils (25 days) are shown at different magnification (the scale bar represents 100 nm) and characterized by ATR-FTIR. Absorbance spectra of the amide I region (thick line) showing the component bands (thin lines). The sum of individual spectral components after Fourier self-deconvolution closely matches the experimental data.

The macromolecular morphological features of PSMs peptide solutions were analyzed using transmission electron microscopy (TEM). In good agreement with the recorded spectral data, ordered fibrils were already observable at 6 days for α-PSM4 and to a lesser extent also for α-PSM1 (Fig. 3a). Both peptide solutions exhibited a large number of unbranched and long amyloid-like fibrils after 25 days. In the case of α-PSM1 they seem to consist mostly of single filaments, whereas for α-PSM4 the filaments tend to associate laterally and twist (Fig. 3b). Conversely, α-PSM2, α-PSM3 and δ-toxin solutions only exhibited a small number of amorphous aggregates or short protofibrilar structures at any assayed incubation time. The secondary structure content of α-PSM1 and α-PSM4 fibrils was analyzed by Attenuated Total Reflectance-Fourier Transform Infrared spectroscopy (ATR-FTIR) (Fig. 3b). Deconvolution of the absorbance spectra in the amide I region allows to analyze the individual secondary structure components and their relative contribution to the main signal (Table 2). The Intense signals between 1615–1626 cm^−1^ for α-PSM1 and α-PSM4, are indicative of intermolecular β-sheets packed into the characteristic amyloid structure^29^. α-PSM1 and α-PSM4 also display common bands at ≈1636 cm^−1^ attributed to intramolecular β-sheet structure and a band at ≈1667 cm^−1^, that may correspond to β-turns or to traces of TFA in the solution. A band at ≈1652 cm^−1^ typically assigned to helical/random conformations is also detected. This may reflect a certain equilibrium between residual helical soluble states and a predominant aggregated assembly. The absence of a split high frequency β-sheet signal at ≈1690 cm^−1^, characteristic of anti-parallel β-sheets, suggests that in α-PSM1 and α-PSM4 peptides the β-sheets are packed in the fibrils in a parallel fashion.

Overall, these data confirm the previously described PSMs ability to form amyloid structures^15^ indicating, however, that this property is only attributable to α-PSM1 and α-PSM4 peptides, which contribute to amyloid formation with distinct aggregation propensities.

### PSMs amyloid fibrils seeding and cross-seeding activity

Amyloid fibril polymerization is rate-limited by the formation of an initial nucleus. The addition of preformed fibrils usually allows to bypass this limitation, enhancing the rate of fibril formation in a phenomenon defined as seeding. Sequence specificity in the seeding of protein amyloid fibrils has been demonstrated^30^, suggesting that protein aggregation can be nucleated only or preferentially by fibrils that share sequence similarity with the soluble species.

**Figure 4:**
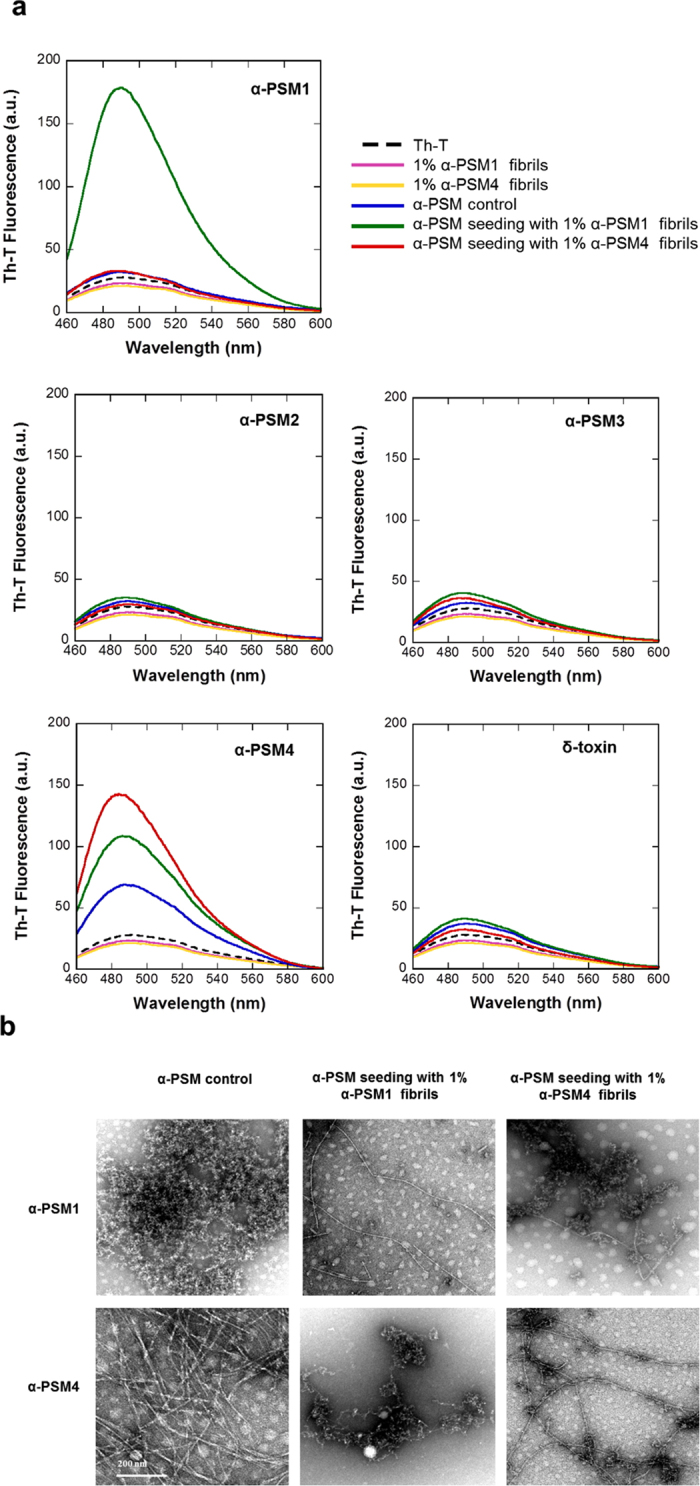
Seeding and cross seeding test. (**a**) PSM seeding and cross-seeding Th-T emission spectra recorded after 6 days of incubation are reported. In all the cases, Th-T free is represented by a black dotted line. (**b**) TEM micrographs of α-PSM1 and α-PSM4 in absence (α-PSM control) and in presence of α-PSM preformed fibrils are shown. The scale bar represents 200 nm.

Despite α-PSM2, α-PSM3 and δ-toxin exhibit negligible intrinsic aggregation propensities, they share significant sequential identity to α-PSM1 and α-PSM4, and is still conceivable that these amyloidogenic variants would seed their aggregation, facilitating their incorporation in the amyloid matrix of the biofilm. To address this possibility seeding and cross-seeding experiments were carried out. PSMs were incubated at 200 μM in their initial soluble states in the absence and in the presence of 1% (v/v) preformed α-PSMs fibrils and amyloid formation was analyzed by measuring Th-T fluorescence after 6 days of incubation (Fig. 4a). No increase in fluorescence was detected for seeds alone throughout the duration of the experiment. The presence of preformed homologous amyloid fibrils induced a large increase in Th-T maximum fluorescence emission compared to control peptide solutions, for both α-PSM1 and α-PSM4. A cross-seeding effect was observed only for α-PSM1 which, when added to a α-PSM4 solution, promoted its aggregation, increasing Th-T fluorescence. However, analysis of α-PSM1 and α-PSM4 seeded and cross-seeded solutions by TEM (Fig. 4b) evidenced that mature well-structured amyloid-like fibrils are formed only in homologous seeding reactions. This suggests that the presence of heterologous fibrils can increase the formation of initial β-sheet enriched protofibrillar aggregates able to bind Th-T, but only a strict sequential identity allows an ordered incorporation of the monomers on top of the seeds.

Overall, we confirm that only α-PSM1 and α-PSM4 display a significant amyloid propensity and that promiscuous cross-seeding does not confer amyloidogenic potential to the rest of PSMs.

### α-PSMs amyloid disaggregation and aggregation in the presence of EGCG

**Figure 5:**
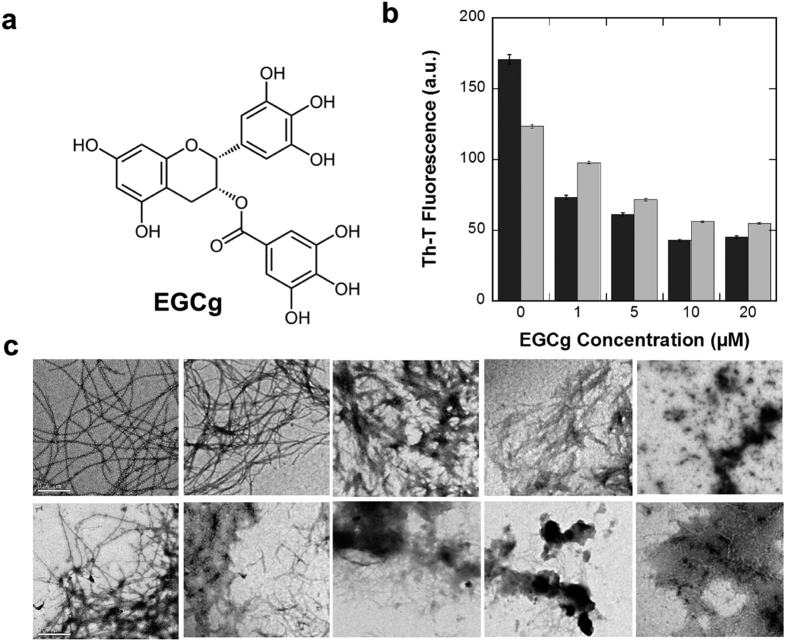
Disaggregation kinetics of α-PSM1 and α-PSM4 fibrils by EGCg. (**a**) Chemical structure of EGCg. (**b**) Disaggregation of α-PSM1 (grey) and α-PSM4 (black) mature fibrils were followed by Th-T emission fluorescence at different EGCg concentration; error bars indicate ± SE (n = 2). (**c**) TEM micrographs of α-PSM1 and α-PSM4 preformed fibrils are displayed in the upper and bottom panels respectively, in presence of 0, 1, 5, 10 and 20 μM of EGCg (from left to right). The scale bar represents 0.5 μm.

Epigallocatechin-3-gallate (EGCG) (Fig. 5a) is the most abundant polyphenolic catechin of the green tea composing more than 50% of its leaves^31^. It has been shown that EGCG inhibits *Staphylococcus epidermis* biofilm formation interfering with the extracellular polymeric material^32^. Interestingly, EGCG has also emerged as a potent inhibitor of Aβ peptide and α-synuclein amyloidogenesis *in vitro* with potential therapeutic application for Alzheimer’s and Parkinson’s diseases^33^.

To test if the reported activity of EGCG on biofilm formation might be mediated by an anti-amyloidogenic activity affecting PSMs assembly, we first tested its ability to disaggregate α-PSM amyloid fibrils. α-PSM1 and α-PSM4 preformed fibrils solutions containing 50 μM Th-T were incubated for 10 min in the presence of different EGCG concentrations. A clear dose dependent EGCG disaggregation effect was observed for both α-PSM fibrils, as revealed by the significant decrease in the dye fluorescence emission signal upon incubation with the compound (Fig. 5b). This property was corroborated by TEM. The long and ordered amyloid fibrils observed in the control solution (without EGCG) were progressively disentangled and converted into amorphous aggregates as the EGCG concentration in the solution increased (Fig. 5c).

**Figure 6:**
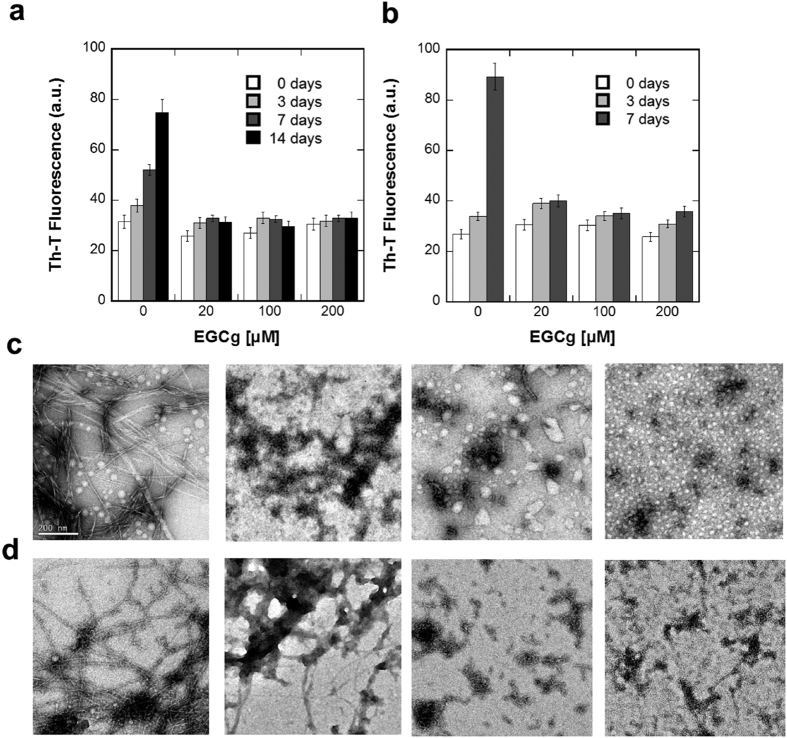
EGCg inhibition effect upon α-PSM1 and α-PSM4 aggregation kinetics. Th-T kinetics of α-PSM1 (**a**) and α-PSM4 (**b**) peptide solutions in presence of EGCg were monitored at different time points. Error bars indicate ± SE (n = 2). Inhibitory effect of EGCg upon α-PSM1 (**c**) and α-PSM4 (**d**) fibril formation at final point was also tested by TEM at 0, 20, 100 and 200 μM EGCg (from left to right). The scale bar represents 200 nm.

Next, to study if apart from a fibril disruptor effect, EGCG possessed also an amyloid inhibitory activity, α-PSM1 and α-PSM4 peptide solutions were prepared at 200 μM in the absence and in the presence of 20, 100 and 200 μM of EGCG and the solutions were incubated for up to two weeks (Fig. 6).

The impact of EGCG on α-PSM1 and α-PSM4 aggregation kinetics was followed by monitoring the Th-T fluorescence emission signal at different time points. For both peptides, EGCG abrogated amyloid formation at all tested concentrations. TEM morphological analysis of α-PSM1 and α-PSM4 solutions confirmed the inhibitory potential of EGCG, as evidenced by the absence of long ordered fibrils and the presence of small size aggregates in the presence of the compound (Fig. 6c,d).

### Modelling α-PSM solubility and toxicity in an *Escherichia coli* model system

All PSMs are secreted without a signal peptide, which implies a dedicated transport mechanism. PSMs are secreted through a specific four-component ABC transporter and blockage of this transporter results in the accumulation of PSMs in the cytosol^34^. Cytosolic accumulation of PSMs is accompanied by abnormal cell division and severe damage to the bacterial cytoplasmic membrane. Therefore, blocking PSMs export has emerged as a potential therapeutic strategy for nosocomial infections, since it will preclude the production of all PSMs simultaneously in addition to directly cause bacterial death^34^. In order to dissect if the toxic effect detected upon intracellular accumulation of PSMs is generic or attributable to specific peptide species and whether this effect is mediated by intracellular aggregates, α-PSM (1–4) peptides were fused to the green fluorescent protein (GFP) and expressed in *Escherichia Coli (E. coli*).

**Figure 7:**
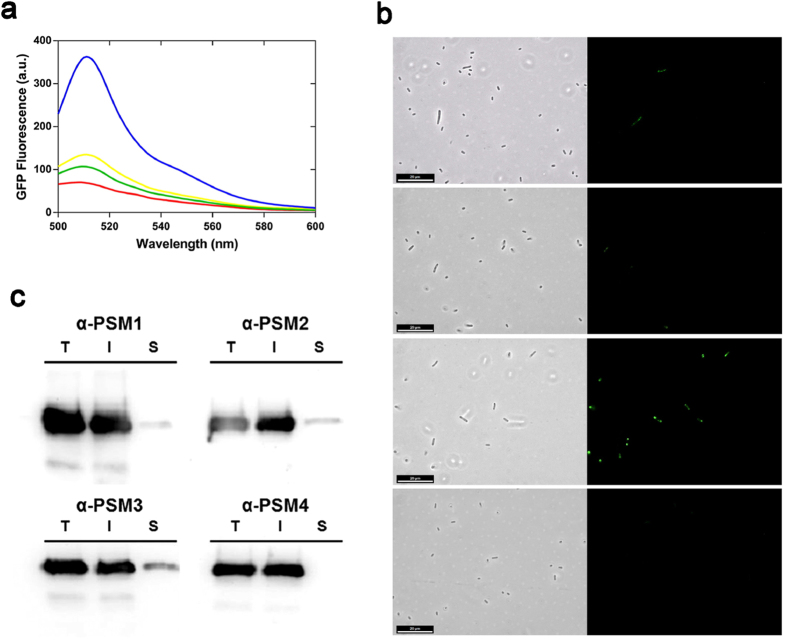
Characterization of α-PSM-GFP fusion protein expression in *E. coli*. (**a**) GFP fluorescence emission spectra of entire cells expressing α-PSM1 (red), α-PSM2 (yellow), α-PSM3 (blue) and α-PSM4 (green) fusions were collected. (**b**) α-PSM-GFP containing cells were observed using fluorescence microscopy (right panels) and phase-contrast microscopy (left panels); the scale bar represents 20 μm. (**c**) Total (T), insoluble (I) and soluble (S) cell fractions of α-PSM fusions were detected by western blotting.

First, the relative intracellular solubility of the peptides was measured by exploiting a previous approach in which it was demonstrated that bacterial GFP fluorescence inversely correlates with the aggregation propensity of the GFP fusion protein partner^35^. The GFP fluorescence emission of intact cells expressing α-PSM-GFP fusion proteins was measured by spectrofluorimetry at 8 h after induction (Fig. 7a). In good agreement with computational predictions, a strong GFP emission signal was observed in cells expressing the α-PSM3-GFP fusion protein, while the fluorescence emission of cells expressing the rest of α-PSM-GFP fusion proteins was much lower. Fluorescence and phase contrast microscopy was used to identify the cellular location of the detected GFP emission (Fig. 7b). In all cases, the presence of inclusion bodies (IBs) was observed by phase contrast microscopy and most of the fluorescence was confined in these aggregates. However, α-PSM3-GFP IBs were clearly more fluorescent than those formed by the rest of α-PSM-GFP, consistent with a lower intracellular α-PSM3 aggregation propensity^36^,^37^.

α-PSM-GFP expressing cells were lysed and the distribution of the fusions between the soluble and insoluble cellular fractions assayed by western blotting using and anti-GFP antibody (Fig. 7c). In all cases, a large majority of α-PSM-GFP fusion proteins were located in the insoluble fraction. In line with their relative predicted aggregation propensities α-PSM4-GFP was totally absent in the soluble fraction, whereas α-PSM3-GFP was the most abundant protein fusion in this fraction.

**Figure 8:**
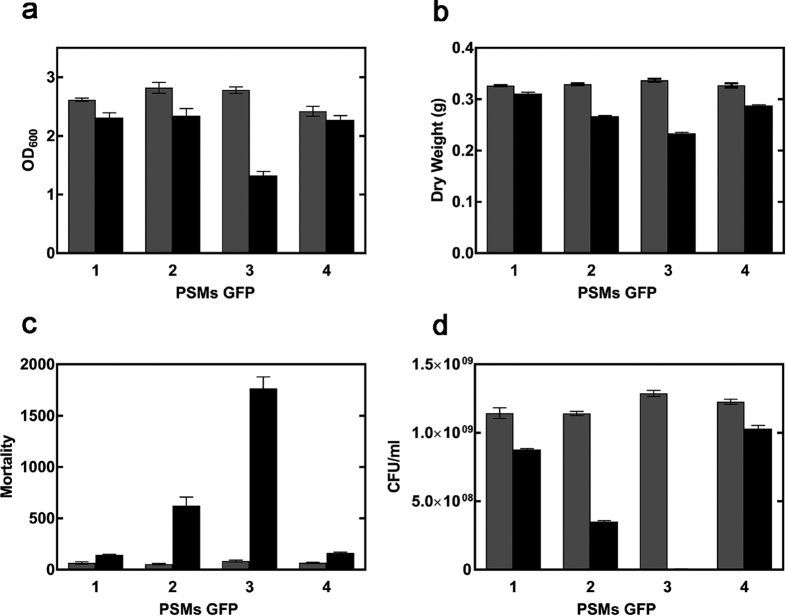
Impact of α-PSMs on cell viability. (**a**) α-PSM fusion cells growth in presence (black) and in absence (grey) of IPTG was analyzed measuring OD600 after 8 h of expression. Error bars indicate ± SE (n = 3). (**b**) Cell density was determined by calculating the dry weight of the α-PSM fusion cells. Viability of *E. coli* expressing α-PSMs was evaluated by (**c**) IP staining and (**d**) CFU counts. Error bars indicate ± SE (n = 3). Bars labelled as 1, 2, 3 and 4 correspond to cells expressing α-PSM1, α-PSM2, α-PSM3 α-PSM4 fusions to GFP, respectively.

In order to identify the peptides responsible for the previously reported intracellular toxicity of PSMs^34^, cells expressing α-PSM-GFP fusion proteins were incubated at 37 °C for 8 h in the presence and absence of IPTG and their growth monitored spectrophotometrically measuring the optical density at 600 nm (OD_600_). As shown in Fig. 8a, α-PSM3-GFP expressing cells exhibited the largest reduction in OD_600_ relative to non-induced control cells. Consistently, the largest decrease in dry cell weight, relative to non-induced control cultures, was also observed for cultures expressing α-PSM3-GFP (Fig. 8b). The viability of control and induced cells was assessed after 8 h using propidium iodide (PI), a dye that only penetrates bacteria with damaged membranes, and flow cytometry. As can be seen in Fig. 8c, expression of α-PSM3-GFP results in a large increase in cell mortality. α-PSM2-GFP expression exerts moderate toxicity, whereas the expression of α-PSM1-GFP and α-PSM4-GFP is virtually innocuous. To confirm this peptide toxicity ranking, we determined the recovery of viable cells by counting the colony forming units (CFUs) upon 8 h in non-induced and induced cell cultures (Fig. 8d). In excellent agreement with flow cytometry data, expression α-PSM1-GFP and α-PSM4-GFP had low impact on cells viability. In contrast, expression of α-PSM3-GFP virtually abrogated the presence of viable cells, reducing them in more than 99.5% relative to its non induced control. The expression of α-PSM2-GFP also reduced significantly the proportion of viable cells.

These data discard intracellular α-PSM-GFP aggregation as the origin of their toxic effect in this model system, since the most amyloidogenic peptides *in vitro* are the most innocuous inside the cell. To confirm this view, the most and less aggregation prone α-PSM4-GFP and α-PSM-GFP3 fusion proteins were expressed at 37 and 25 °C and the GFP fluorescence of intact cells (Fig. S1) and cultures cell densities (Fig. S1) were analyzed. In both cases, a significant increase in fluorescence emission signal was observed at 25 °C, consistent with an increase in the solubility of the fusion at low temperatures, as we have shown for other GFP fusions to aggregation-prone proteins^38^. However, only α-PSM3-GFP expression impacts significantly cell density, independently of the assayed temperature, confirming that the PSMs intracellular toxic effect is not associated with the generic PSMs solubility.

## Discussion

PSMs peptides are major determinants of *S. aureus* virulence^17^. α-PSMs are the smallest staphylococcal toxins belonging to PSM family. They have been well characterized because their immune-modulating and their cytolytic activity^39^,^40^. Recently, different studies have reported their involvement in biofilm formation and detachment, suggesting new α-PSMs functions in staphylococcal pathogenesis^41^,^42^. Interestingly enough, α-PSMs seem to act forming fibrillary amyloid structures that sustain the integrity of the *S. aureus* biofilm^15^. These amyloid-like structures are conceptually analogous to those being found in the biofilms of an increasing number of bacteria^13^. In the present work, we analyzed the *S. aureus* PSMs (including δ-toxin) amyloid formation mechanism in order to dissect the specific contribution of these short peptides to the biofilm structure and how this relates to intracellular toxicity.

Computational analysis of α-PSMs sequences already suggested that the peptides in this family might display differential self-assembly properties with α-PSM4 and α-PSM3 predicted as those having the longest and shortest aggregation-prone regions, respectively (Table 1). These differences were confirmed *in vitro* demonstrating that, contrary to what was previously thought^15^, not all PSMs form amyloid structures. According to our data, α-PSM1 and α-PSM4 peptides would be the major contributors for the reported α-PSMs fibrillogenesis (Fig. 2a). α-PSM1 and α-PSM4 aggregates display canonical fibrillar morphology, bind to amyloid dyes, are enriched in intermolecular β-sheet conformation and seed the aggregation of freshly homologous peptide solutions. EGCG, the principal polyphenol present in green tea, has been shown to be effective at preventing aggregation and is able to remodel amyloid fibrils comprising different amyloidogenic proteins^33^,^43^,^44^. We show here that EGCG is also active against α-PSM1 and α-PSM4 amyloid fibrils, which suggests its potential application to disrupt or weaken the amyloid matrix in *S. aureus* biofilms. Besides, α-PSM expression in *E. coli* revealed that the intracellular aggregation of α-PSM1 and α-PSM4 peptides does not perturb cell fitness, which is consistent with a functional role of these peptides in *S. aureus* biofilm polymerization, a process in which they are expected to be innocuous.

The *S. aureus* α-PSM transporter (pmt) is a promising therapeutic target because its inactivation would prevent the translocation of PSMs peptides to the extracellular space, causing their cytoplasmatic accumulation, resulting in a loss of bacterial fitness^34^. Our results argue that the deleterious impact of intracellular accumulation of α-PSMs in *S. aureus* upon blockage of their secretion would be essentially exerted by the more soluble α-PSM3, and not by α-PSM1 and α-PSM4, that would likely aggregate into inert inclusions. Interestingly, when the α-PSMs accumulated in the cytosol of a pmt deletion mutant were analyzed, α-PSM2, α-PSM3 and δ-toxin could be identified, but not α-PSM1 and α-PSM4^34^. It was proposed that this absence would owe to lower production, degradation or nonspecific adhesion to cellular material, but our results strongly suggest that they were not detected because they accumulated as insoluble aggregates and therefore they were not present in the analyzed cellular supernatant^34^.

All PSMs are assumed to share a conserved native α-helical structure in their physiological context. Their lytic activity is thought to be somehow associated with the hydrophobic character of the α-helical motif, since hydrophobic residues are expected to promote PSMs aggregation, concomitantly decreasing the amount of active available peptide. This will decrease the peptide interactions with membranes and their disruptive effect^45^,^46^, in a mechanism analogous to that described for α-helical antimicrobial peptides (AMPs)^47^,^48^. In order to understand the “weight” of hydrophobicity in the α-helical structure of α-PSMs we analyzed their sequences with JPRED4^49^ to predict the stretch of amino acidic residues forming the α-helix and then represented them using helical wheel projections (Fig. S3). Next, we calculated the hydropathy index of the predicted α-helix sequences using the GRAVY value^50^ (Table 3). As expected, α-PSM4 showed the most hydrophobic score (2.01) while α-PSM3 (0.67) resulted to be the less hydrophobic peptide, in good agreement with their distinct functions. Interestingly, calculation of the hydropathy of the residues belonging either to the hydrophobic or hydrophilic halves of the wheel projections, suggested that it is indeed the contribution of residues on the hydrophilic side that modulates the final amyloid ability of the peptides, as indicated by the less negative GRAVY values at the hydrophilic face in α-PSM4 and α-PSM1. This suggests that the spatial distribution of the residues in the different peptides contributes to their different amyloid propensity. Importantly, our data indicate that the α-helical propensities of these peptides might differ significantly, being likely another important factor that determines their amyloid potential. In this way, α-PSM3 remains for long time in an α-helical conformation, whereas α-PSM4 rapidly transitions towards a β-sheet structure. However, helical propensities alone cannot explain the differences in amyloid potential and toxicity of peptides α-PSM1 and α-PSM2. Overall, our results suggest that PSM functions rely on a fine balance of hydrophobic/hydrophilic forces and α-helical propensity.

## Experimental Procedures

### Peptides and preparation

α-PSM1 (MGIIAGIIKVIKSLIEQFTGK), α-PSM2 (MGIIAGIIKFIKGLIEKFTGK), α-PSM3 (MEFVAKLFKFFKDLLGKFLGNN, α-PSM4 (MAIVGTIIKIIKAIIDIFAK) and δ-toxin (MAQDIISTIGDLVKWIIDTVNKFTKK) peptides were purchased from ChinaPeptides (Shanghai, China) with a purity >95%. They were dissolved to a final concentration of 0.5 mg/mL in a 1:1 mixture of trifluoroacetic acid (TFA) and hexafluoroisopropanol (HFIP). Peptides were then sonicated for 10 minutes and incubated for 1 h at room temperature. Stock solutions were divided into aliquots, solvent TFA/HFIP dried with a SpeedVac (Thermo Scientific, USA) at room temperature and stored at −80 °C. All assays were performed with equal stoichiometric ratios of peptides unless otherwise described. When required, samples were resuspended in anhydrous dimethyl sulfoxide (5%) and sonicated for 10 minutes. Sonication was crucial for removing any trace of undissolved seeds that may resist solubilization. This preparation yielded phenol soluble modulins in monomeric form. Peptide aliquots were prepared in MilliQ water yielding a final peptide concentration of 200 μM.

### Prediction of amyloid sequence stretches and aggregation propensities

PSMs peptides aggregation-prone sequence stretches prediction was performed using the Amylpred2^28^ aggregation prediction algorithm employing simultaneously the eleven independent predictors incorporated in this software.

### Thioflavin-T binding

200 μM peptide solutions were incubated at room temperature. Peptide aggregation was monitored by measuring the transition from the non-aggregated to the aggregated state by relative Th-T (50 μM) fluorescence emission at 490 nm upon excitation at 440 nm. In the seeding and cross-seeding assays, 1% (v/v) of preformed aggregates were added to peptide solution and incubated during 6 days at room temperature. For aggregation monitoring, 50 μM Th-T dye was added to 10 μM peptide solution. Spectra were recorded as the accumulation of three consecutive scans between 460 and 600 nm with an excitation wavelength of 440 nm on a Jasco FP8200 spectrofluorometer (Jasco, Japan). For all measures, a 5 nm slit width was used for both excitation and emission.

### Congo red binding

Congo red (CR) binding with aggregated peptides was analyzed in the range 400–700 nm using a 1 cm optical length quartz cuvette placed in a thermostated cell holder at 25 °C on a Cary-400 UV/Vis spectrophotometer (Varian Inc., USA). 20 μM CR was mixed with 12 μM peptide sample solution.

### Attenuated total reflectance (ATR) fourier transform infrared (FTIR) spectroscopy

ATR-FTIR analysis was performed using a Bruker Tensor 27 FTIR Spectrometer (Bruker Optics Inc., USA) with a Golden Gate MKII ATR accessory. Aggregated peptide solutions were dried out under a N_2_(g) atmosphere and each spectrum was measured as the accumulation of 16 scans at a spectral resolution of 2 cm^−1^ within the range 1800–1500 cm^−1^. The individual components of the spectrum were determined through second derivative analysis of the spectra and deconvoluted afterwards into overlapping Gaussian curves by employing the non-linear fitting program PeakFit v4.12 (Systat Software Inc., USA).

### Circular dichroism analysis

Far-UV CD spectra were measured in a Jasco-710 spectropolarimeter (Jasco, Japan) thermostated at 25 °C. Aggregated peptides were prepared at 20 μM and measured immediately. Spectra were recorded from 260 to 200 nm, at 0.5 nm intervals, 1 nm bandwidth, and a scan speed of 100 nm/min. For each spectrum twenty accumulations were averaged.

### Transmission electron microscopy (TEM)

Fibrillar peptide solutions were resuspended in water, placed on carbon-coated copper grids, and left for 5 min. The grids were then washed twice with distilled water, stained with 2% (w/v) uranyl acetate for a minute, and dried out before analysis. TEM JEM-1400 microscope was used operating at an accelerating voltage of 120 kV.

### Epigallocatechin gallate (EGCG) disaggregation and inhibition assays

To test the effect of EGCG disaggregation, 5% v/v preformed fibrils were added to 50 μM Th-T solution and incubated with 0, 1, 5, 10 and 20 μM EGCG (99% pure; Sigma, USA). After 10 min of incubation Th-T fluorescence emission at 490 nm was recorded for each sample. Solutions for peptide aggregation kinetics were prepared at 200 μM in the presence of 0, 10, 20 and 200 μM of EGCG and amyloid formation monitored as described in the previous section.

### Cloning and expression of PSM-GFP fusions

Single stranded oligonucleotides (ssDNA) corresponding to the PSM peptides (α-PSM1, α-PSM2, α-PSM3, α-PSM4) were purchased from Invitrogen (Thermo Fisher Scientific Inc., USA). PSM ssDNAs were phosphorylated and annealed to obtain double stranded oligonucleotides (dsDNAs). A pET28a vector already containing a fusion of Amyloid β-42 (Aβ-42) peptide with the Enhanced Green Fluorescent Protein (GFP)^49^ was engineered by PCR reaction to remove Aβ-42 gene and introduce the PSM insert upstream of the GFP sequence finally encoding an N-terminal PSM-GFP fusion protein. All constructs were verified by DNA sequencing. Plasmids were then transformed into *Escherichia coli* BL21 (DE3) cells and cultures were grown in Luria Broth (LB) medium, containing appropriate antibiotics, with agitation (250 rpm) at 37 °C (25 °C when needed). Protein expression was induced at an OD_600_ of 0.5, with 1 mM isopropyl β-D-1-thiogalactopyranoside (IPTG) for 8 h.

### Cell optical density (OD) and fluorescence measurements

Cell cultures induced for 8 hours were harvested at 3,000 rpm and washed in PBS for three times. Optical density at 600 nm (OD_600_) was recorded for each sample on a Cary-400 UV/Vis spectrophotometer (Varian Inc., USA). GFP fluorescence of intact cells at an OD_600_ = 0.1 was recorded in the range of 500–600 nm, using an excitation wavelength of 470 nm on a Jasco FP8200 spectrofluorometer (Jasco, Japan). For microscopic analysis, 10 μL of washed intact cells were deposited on top of glass slides and imaged with a phase-contrast and fluorescence microscopy under UV light using a Leica Q500 MC fluorescence DMBR microscope (Leica Microsystems, Germany).

### Viable bacterial cell account

Determination of the viable bacterial count was assessed by serial dilutions of the cell cultures induced for 8 h at 37 °C, and the number of colonies was counted as a colony-forming unit (CFU).

### Dry weight measurement

The cell density was also quantified by dry weight assessment. 150 ml of the cell cultures induced for 8 h, were harvested by centrifugation at 10,000 rpm, 4 °C for 15 minutes and washed twice with ice-cold PBS. The bacteria were transferred to pre-weighted tubes and were dried at 70 °C for 24 h in a hot air oven. The mass was recorded on a four-place balance (Mettler Toledo AJ 100, USA).

### Propidium iodide staining and flow cytometry

Induced *E. coli* cell cultures expressing α-PSMs were washed with filtered PBS and diluted to an OD_600_ of 0.2. Then samples were stained with propidium iodide (PI) dye (BD Biosciences) to a final concentration of 50 μM. As a positive control 1 mL of sample was heated at 90 °C for 10 min and stained with PI. Flow cytometry was performed using a BD FACSCanto flow cytometer (BD Biosciences) equipped with 488 nm and 635 nm lasers. Bacterial samples were gated for side scatter (SCC) vs forward scatter (FSC) in such a way that cell debris and non-intact cells were excluded from the analysis. The analyzed data correspond to PE vs FSC channels with a threshold set up with non-stained cells. We acquired a total of 20,000 events logarithmically (five decades). Data were acquired with the FACSDiva Software (BD Biosciences). Data analysis was performed with the FlowJo software. All experiments were performed using three biological replicates.

### Western blotting

Cell pellets were resuspended in PBS at an OD_600_ = 2. Samples were sonicated and centrifuged at maximum speed for 20 min at 4 °C. The soluble, insoluble and total fractions were resolved on 12% w/v SDS-PAGE gel and transferred onto a PVDF membrane. Immunodetection was performed using anti-GFP antibody (BD Biosciences) and membranes were developed with ECL Chemiluminescent HRP Substrate (Millipore) according to the manufacturer’s protocols. Densitometry bands analysis was performed using the Image J software.

## Additional Information

**How to cite this article**: Marinelli, P. *et al*. Dissecting the contribution of *Staphylococcus aureus* α-phenol-soluble modulins to biofilm amyloid structure. *Sci. Rep.* **6**, 34552; doi: 10.1038/srep34552 (2016).

## Supplementary Material

Supplementary Information

## Figures and Tables

**Table 1:** Predicted aggregation regions of PSM peptides using Amylpred2^24^ and percentage of amino acids in those regions relative to the total length of the peptide.

	α-PSM1	α-PSM2	α-PSM3	α-PSM4	δ-toxin
Amyloidogenic regions	3–14	3–16	6–8	1–19	4–9, 11–19
Amyloidogenic sequence (%)	57.1	66.7	13.6	90.0	60.0

**Table 2:** Secondary structure bands in the deconvoluted absorbance FTIR spectra of α-PSM1 and α-PSM4 aggregated peptide solutions.

	Bands (cm^−1^)	% Area	Structure
α-PSM1	1624	38.5	β-sheet (inter)
	1634	18.5	β-sheet (intra)
	1651	28.8	α-helix/random
	1667	14.2	turn/TFA traces
α-PSM4	1615/1626	43.2	β-sheet (inter)
	1638	16.5	β-sheet (intra)
	1652	28.2	α-helix/random
	1667	12.1	Turn/TFA traces

**Table 3:** α-PSM hydropathy.

	Sequence^a^	T. GRAVY^b^ value	P. GRAVY^c^ value	P. GRAVY^d^ value
**α-PSM1**	GIIAGIIKVIKSLIEQF	1.36	4.2	−1.15
**α-PSM2**	GIIAGIIKFIKGLIEKF	1.28	4.0	−1.82
**α-PSM3**	EFVAKLFKFFKDLLGKFL	0.67	3.3	−2.65
**α-PSM4**	AIVGTIIKIIKAIIDIF	2.01	4.3	−1.25

The GRAVY value is calculated by adding the hydropathy values^50^ of each amino acid residue and dividing it by the number of residues in the predicted helical sequence. Increasing positive scores indicate a greater hydrophobicity.

^a^α-PSM sequence represented as wheel projection in Fig. S1.

^b^GRAVY value calculated for the fully α-helical structures predicted by JPRED4 server in a.

^c^GRAVY value calculated for the residues (underlined in the sequence^a^) belonging to the hydrophobic half of wheel projection in Fig. S1.

^d^GRAVY value calculated for the residues belonging to the hydrophilic half of wheel projection in Fig. S1.
