# Supplementary material for: Dissecting the contribution of Staphylococcus aureus α-phenol-soluble modulins to biofilm amyloid structure
Source: Sci Rep. 2016 Oct 6;6:34552. doi: 10.1038/srep34552 (PMC5052566; doi:10.1038/srep34552)
Supplement: Supplementary Information [file srep34552-s1.pdf]

Scientific Reports

Dissecting the contribution of *Staphylococcus aureus* α-phenol-soluble modulins to biofilm amyloid structure

Patrizia Marinelli[1], Irantzu Pallares[1,2], Susanna Navarro[1,2] and Salvador Ventura[1,2¶]

[1]Institut de Biotecnologia i de Biomedicina
[2]Departament de Bioquímica i Biologia Molecular. Universitat Autònoma de Barcelona. E-08193 Bellaterra (Spain).

SUPPLEMENTARY INFORMATION:

Table S1: Secondary structure content in the deconvoluted CD spectra of freshly dissolved PSM peptides using the CONTIN algorithm.

Supplementary Figure S1. Effect of temperature on α-PSMs solubility and cell density.

Supplementary Figure S2: Helical wheel projections of PSMs.

Table S1 Secondary structure content in the deconvoluted CD spectra of freshly dissolved PSM peptides using the CONTIN algorithm[(28)]

Structure	α-PSM1	α-PSM2	α-PSM3	α-PSM4	δ-toxin
**α-helix (%)**	32.1	30.4	68.1	49.1	72.5
**β-sheet (%)**	5.3	5.7	4.9	15.8	5.6
**Disordered (%)**	62.6	63.9	27.0	35.1	21.9

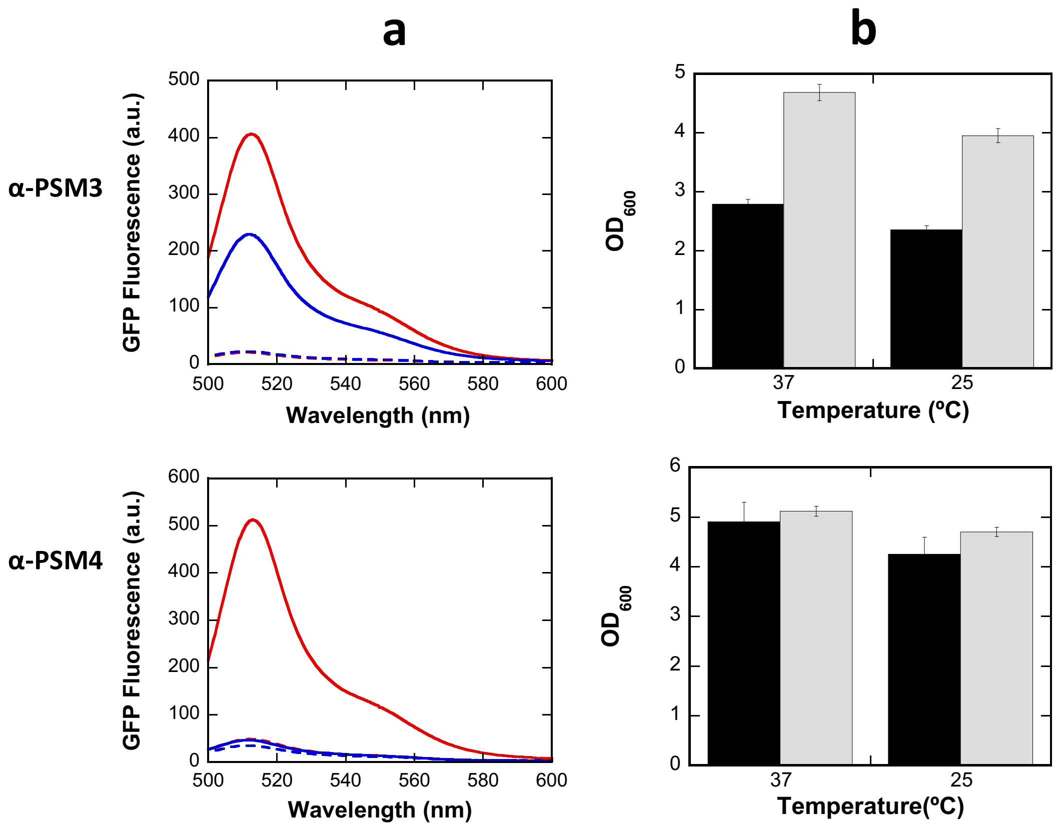

Supplementary Figure S1. Effect of temperature on α-PSMs solubility and cell density. (A) GFP fluorescence was measured for intact cells expressing α-PSM3 and α-PSM4 fusion proteins at 37 °C (blue) and 25 °C (red) in presence (solid line) and in absence (dotted line) of IPTG. (B) OD_{600} of α-PSM3 and α-PSM4 fusion expressing cells grown at different temperature, with (black) and without (grey) IPTG. Error bars indicate ± SE (n= 3).

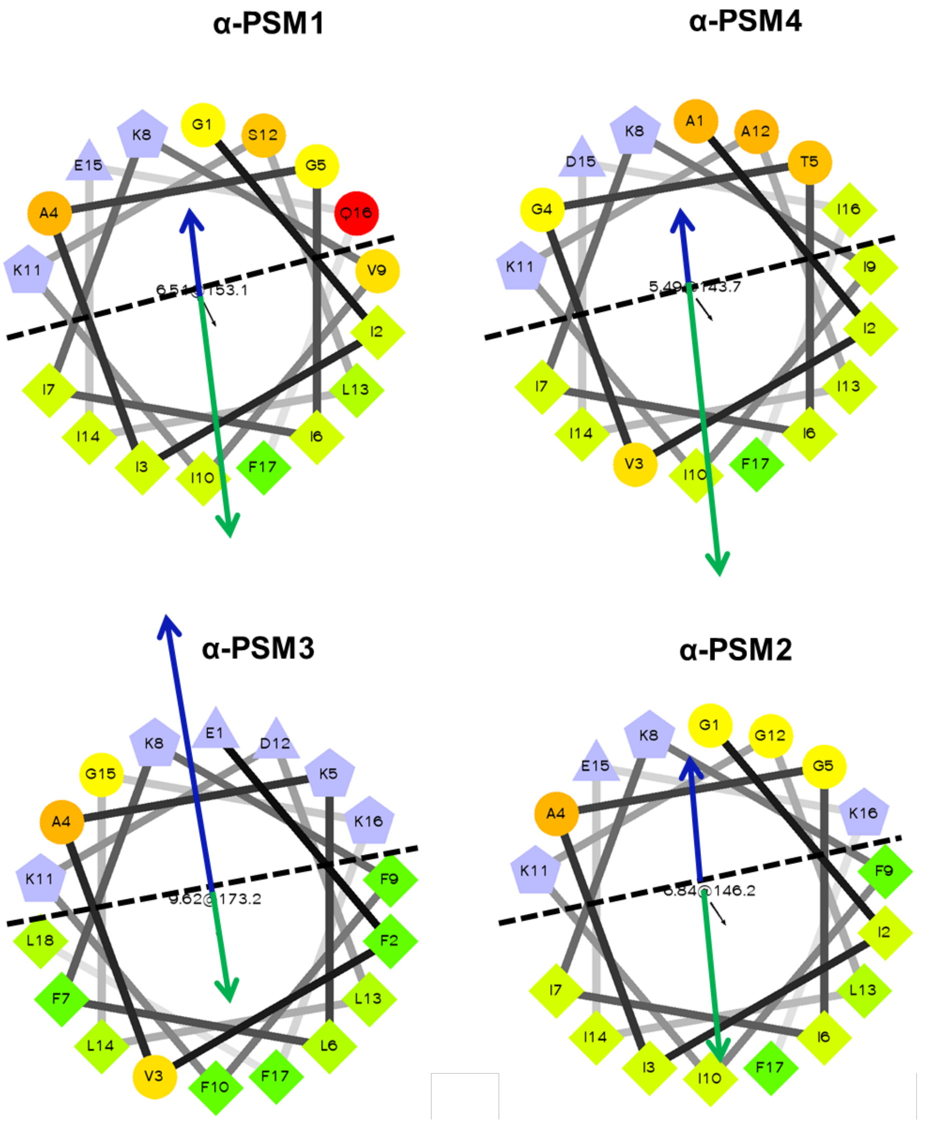

Supplementary Figure S2: Helical wheel projections of PSMs. PSMs sequence forming α-helical structures predicted through JPRED4 server[59] are represented. Hydrophobic residues are color coded from green (most hydrophobic) to yellow (zero hydrophobicity). Hydrophilic residues are coded with pure red (the most hydrophilic), and the amount of red decreasing proportionally to the hydrophilicity; the potentially charged residues are light blue. The resulting hydrophilic and hydrophobic contributions are represented as blue and green arrows, respectively. Helical wheel projections were performed using the Helical Wheel Plotting program by (http://rzlab.ucr.edu/).
